# Anogenital high-risk HPV prevalence and screening considerations in female transplant recipients: a cross-sectional study

**DOI:** 10.1186/s12905-025-03813-0

**Published:** 2025-07-03

**Authors:** Christoph Hillen, Charlotte Sachs, Anna Jaeger, Katharina Prieske, Barbara Schmalfeldt, Marc Lütgehetmann, Martina Sterneck, Malte A. Kluger, Andreas M. Kaufmann, Eik Vettorazzi, Linn Woelber

**Affiliations:** 1 Department of Gynecology and Gynecologic Oncology, University Medical Center Hamburg-Eppendorf, Hamburg, Germany; 2 Dysplasia Center, Jerusalem Hospital, Hamburg, Germany; 3 Hygiene Institute for Medical Microbiology, Virology and Hygiene, University Medical Center Hamburg-Eppendorf, Hamburg, Germany; 4 I. Department of Internal Medicine 1, University Medical Center Hamburg-Eppendorf, Hamburg, Germany; 5 University Transplant Center, University Medical Center Hamburg-Eppendorf, Hamburg, Germany; 6 III. Department of Medicine, University Medical Center Hamburg-Eppendorf, Hamburg, Germany; 7 Department of Gynecology, HPV Research Laboratory, Charité – Universitätsmedizin Berlin, Corporate Member of Freie Universität Berlin and Humboldt Universität zu Berlin, Augustenburger Platz 1, 13353 Berlin, Germany; 8 Institute of Medical Biometry and Epidemiology, University Medical Center Hamburg-Eppendorf, Hamburg, Germany

**Keywords:** Organ transplantation, Immunosuppressive therapy, High-risk HPV prevalence, Cervical HrHPV, Anal HrHPV, Kidney transplant recipients, Liver transplant recipients

## Abstract

**Purpose:**

Organ transplant recipients receiving immunosuppressive therapy have a higher risk of developing anogenital HPV-related (pre)malignancies. This study aims to determine the age-dependent anogenital hrHPV prevalence in transplanted patients and to investigate various risk factors within this high-risk patient group.

**Methods:**

Women (*n* = 201) after kidney (*n* = 98), liver (*n* = 93), and combined (*n* = 10) transplantation were tested and genotyped for hrHPV in the anal and cervical regions. Medical records and a questionnaire assessed sexual behaviour, medical treatment history, and demographic data.

**Results:**

Among our cohort the median age was 52 years (range 18–78), the prevalence of cervical hrHPV infection was 15.9% (32/201), with no significant difference observed between liver and kidney transplant recipients. Increased hrHPV prevalence was not associated with transplant-specific risk factors such as type and duration of immunosuppressive therapy, whereas typical risk factors such as sexual behaviour conferred increased risk. Anal hrHPV had an overall prevalence of 20.3%, and the co-prevalence of hrHPV detected at both anal and cervical sites was 68.8%. 90% of the patients with cervical hrHPV infection (29/32) attended clinical follow-up and high-grade intraepithelial neoplasia was detected in four women (3x CIN2+/ 1x VaIN3).

**Conclusion:**

Compared to the general population, our study shows an increased cervical HPV prevalence in transplanted patients above 30 years. In addition, the baseline risk for infection with anal hrHPV is increased, suggesting that additional screening in cervical hrHPV-positive transplanted patients may be beneficial to detect (pre)invasive lesions in the anal region.

## Take-home message

Female organ transplant recipients show a high genital hrHPV prevalence with an increased anal co-infection rate, suggesting additional screening for genitoanal lesions might be beneficial.

## Introduction

Persistent infection with high-risk Human Papillomavirus (hrHPV) is a prerequisite for the development of cervical intraepithelial neoplasia (CIN) and cervical cancer. The development of cervical cancer can be largely prevented through vaccination and screening [1]. The progression from infection to cancer usually takes decades, so detecting hrHPV or CIN at an early stage and, if necessary, initiating correct further treatment is highly beneficial for patients. HPV 16 and 18 have high oncogenic potential and are detected in 70% of cervical cancers [2]. Persistent hrHPV infection is also a major cause of anal cancer, with HPV16 playing a pivotal role. There is a strong correlation between cervical and anal HPV infections, so cervical HPV-based screening could improve screening for anal cancer risk by allowing patients with hrHPV positivity in the cervix also to receive screening for anal changes [3].

Transplanted patients receive lifelong immunosuppressive treatment to prevent rejection. However, drug treatment is associated with an elevated risk of developing anogenital (pre)malignancies related to HPV [4–6]. Given the increased incidence of genitoanal dysplasia, one might conclude that hrHPV prevalence is elevated in transplanted women. Nevertheless, previous studies regarding cervical and anal HPV prevalence have shown contradictory results: Reported cervical hrHPV prevalence in transplanted patients varies between 7% and 40% and anal prevalence between 8% and 46% depending on the testing method and study population [7–15]. The wide variation in reported prevalence might also be attributed to the small sample size and locoregional differences.

No screening algorithms beyond the guideline-based cervical cancer screening in the general population are currently recommended for organ transplant recipients (dependent on national programs, hrHPV testing from 30 to 35 years and cervical cytology is recommended) [16].

Early detection of high-grade squamous intraepithelial lesions (HSIL) is critical for the prevention of anal carcinoma, as the treatment of HSIL significantly reduces the risk of progression to anal cancer [17].To reduce the risk of anal cancer in transplanted patients, the International Anal Neoplasia Society (IANS) currently recommends screening through a digital rectal examination combined with anal cytology and/or HPV testing, beginning 10 years after transplantation [18]. An abnormal finding should then be followed by high-resolution anoscopy.

This study aims to determine the age-dependent genitoanal hrHPV prevalence in liver and kidney transplant recipients and to identify possible risk factors for HPV infection.

## Methods

### Study population

In this cross-sectional study, female patients who received kidney or liver transplantation were eligible for participation if they met the following criteria: (1) minimum age of 18 years, (2) time interval to organ transplantation of at least 12 months, (3) treatment at the Transplant Center of the university hospital. Patients with hysterectomies were excluded. The Hamburg Medical Association Ethics Committee approved the study in June 2019 (Ethics vote: PV6011). Patients were informed by telephone, in writing, or verbally at the transplant centre about possible study participation. After written informed consent was obtained, patients completed a standardized questionnaire, and cervical and anal swabs were collected. Medical records were used to gather information regarding medication and co-morbidities. All eligible patients, who were willing to participate in the study were enrolled between November 2019 and July 2021.

### HPV-testing

Cervical and anal swab collection was performed by a gynecologist or trained personnel under supervision. The cervical swabs were collected with the Rovers Cervex-Brush and preserved in Roche Cell Collection Medium. For the anal swab, the Bruker eSwabTM system or Mantacc’s MBT-010 Virus Transport Medium was used for preservation. The cervical specimens were then tested with the cobas HPV assay through the cobas 6800 system. This HPV assay detects HPV 16 and 18, and 12 pooled hrHPV types (31, 33, 35, 39, 45, 51, 52, 56, 58, 59, 66, 68). If the test result was positive, the patients were informed. Further cervical and anal HPV testing were analyzed by Seegene’s AnyplexTM II HPV28 Detection – System for verification and genotyping as this is the standard test in our center. This allows the simultaneous detection of 19 hrHPV types (HPV 16, 18, 26, 31, 33, 35, 39, 45, 51, 52, 53, 56, 58, 59, 66, 68, 69, 73, 82) and low risk (lr)HPV (HPV 6, 11, 40, 42, 43, 44, 54, 61, 70). In this study, only the detection of hrHPV was investigated, and lrHPV was not considered in the statistical analysis. Testing of anal swabs for HPV-DNA is not validated by the manufacturer regarding clinical sensitivity and specificity and is performed as a research-use-only assay.

### Follow-up

Patients who tested positive for hrHPV in the cervical area were readmitted to our dysplasia clinic, receiving cytology, colposcopy, and, if necessary, a biopsy of suspicious lesions. Follow-up was performed between 6 and 18 months after initial testing. Since the cobas HPV assay only provides pooled genotyping, HPV testing in the cervical and anal regions was repeated in cobas positive patients, and further genotyping was performed using Seegene’s Anyplex™ assay. Patients who tested HPV positive only in the anal region were informed of the result and recommended follow-up with a practicing proctologist. HPV negative patients did not receive follow-up.

### Questionnaire

All study participants were asked to complete a questionnaire created for this study (see supplements). This questionnaire included baseline data like age, type of transplantation, and medical history, focusing on immunosuppression, comorbidities, and sexual history. Where possible, we cross-checked patient information with hospital records to ensure accuracy and to fill in any missing information in the questionnaire.

### Data and statistical analysis

Descriptive statistics were presented as medians and ranges for continuous data and counts or percentages for discrete data. Groups were compared using the Kruskal-Wallis rank sum test for continuous data and Fisher’s exact test for discrete data. To evaluate risk factors of hrHPV infection we performed a multiple logistic regression analysis and included smoking, age and type of transplantation as potential confounders. Explanatory variables were selected based on clinical relevance. Multicollinearity was assessed using Variance Inflation Factors (VIF), all of which were below 3. We did not test for interactions among the independent variables included due to limited sample size and lack of theoretical justification. An alpha level of 0.05 was used for all statistical tests.

Statistical analysis was performed using R (version 4.0.5, Windows, R core group, Vienna, Austria, 2021). Missing answers were excluded from the analysis. In this study, the duration of immunosuppression refers to the time since the first transplant (for multiple transplant recipients). Graph Pad Prism was used to create figures.

## Results

Between November 2019 and July 2021, 201 women were enrolled in the study. These included 98 kidney transplant recipients, 93 liver transplant recipients, and ten patients who received simultaneous kidney and liver transplantation. 65.2% of patients knew they were at increased risk for genitoanal cancers, and 77.6% knew cervical screening could reduce cancer risk. In addition, most patients (92.5%) reported regularly taking advantage of the cervical screening offer.

### HrHPV prevalence

**Fig. 1:**
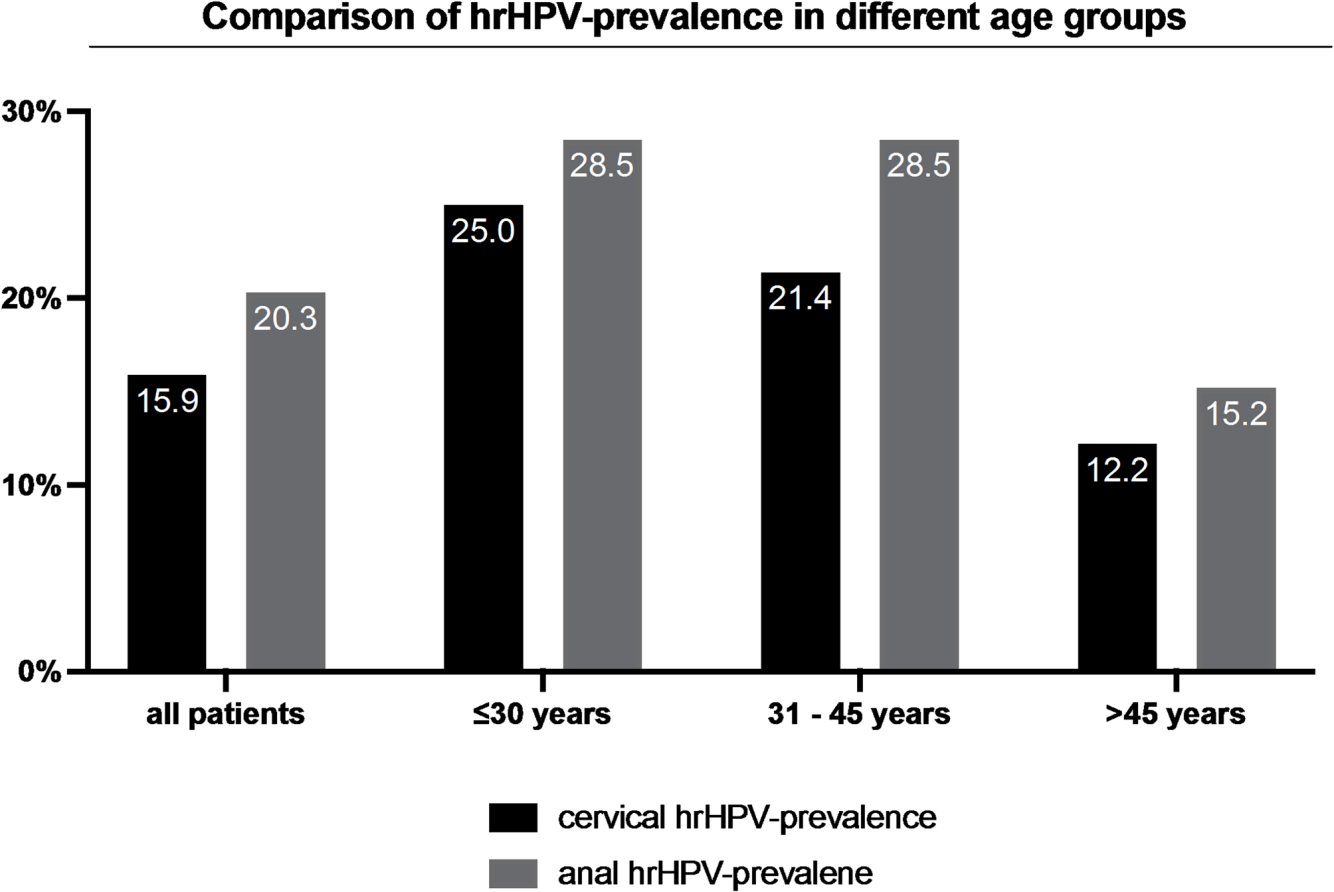
hrHPV = high-risk human papillomavirus

The baseline characteristics of the participants are shown in Table 1, which compares hrHPV positive and negative patients. Overall, 32 out of 201 (15.9%) patients tested positive for hrHPV at the cervical site. The anal hrHPV prevalence was 20.3% (40/197). No significant difference in cervical hrHPV prevalence was found between kidney and liver transplanted patients (see Table 2). The median age was 52 years (median 18–78), with cervical hrHPV-positive patients being, on average, eight years younger (*p* = 0.029). HrHPV prevalence declined with increasing age as shown in Fig. 1. However, the differences in cervical hrHPV prevalence across age groups were not statistically significant (*p* = 0.068), whereas anal hrHPV prevalence did show a significant difference across age groups (*p* = 0.038). When comparing individuals aged ≤ 45 years to those > 45 years, anal hrHPV prevalence was significantly higher in the younger age group (*p* = 0.020). In contrast, cervical hrHPV prevalence showed a non-significant trend towards higher rates in the younger group (*p* = 0.078).The median body mass index (BMI) was 23.4 in the cervical hrHPV-negative group and 21.4 in the cervical hrHPV-positive group (*p* = 0.030).

**Table 1:** Baseline characteristics of the study population comparing cervical hr-HPV-positive and negative patients

	Total (*N* = 201)	Positive (*N* = 32)	Negative (*N* = 169)	*p*-value
**Median Age in years (range)**	52.0 (18.0, 78.0)	44.0 (19.0, 72.0)	52.0 (18.0, 78.0)	*
**Median BMI (range)**	23.2 (16.2, 38.4)	21.4 (18.3, 37.9)	23.4 (16.2, 38.4)	*
Kidneytransplantation	108 (53.7%)	18 (56.2%)	90 (53.3%)	NS
Livertransplantation	103 (51.2%)	15 (46.9%)	88 (52.1%)	NS
Combined transplantation	10 (5.0%)	1 (3.1%)	9 (5.3%)	NS
**Years since first transplantation (range)**	9.0 (1.0, 42.0)	8.5 (1.0, 23.0)	9.0 (1.0, 42.0)	NS
< 5 years	55 (27.4%)	7 (21.9%)	48 (28.4%)	NS
5 - <10 years	47 (23.4%)	10 (31.2%)	37 (21.9%)	NS
10 - <15 years	39 (19.4%)	6 (18.8%)	33 (19.5%)	NS
≥ 15 years	60 (29.9%)	9 (28.1%)	51 (30.2%)	NS
Patient knew about elevated cancer risk	131 (65.2%)	17 (53.1%)	114 (67.5%)	NS
Patient knew about cancer screening	156 (77.6%)	25 (78.1%)	131 (77.5%)	NS
Regularly participates in cancer screening examinations	186 (92.5%)	29 (90.6%)	157 (92.9%)	NS
**Nicotine consumption**	26 (12.9%)	5 (15.6%)	21 (12.4%)	NS
**Consumes alcohol**	73 (36.3%)	16 (50.0%)	57 (33.7%)	NS
(pre)cancerous lesion in history	72 (35.8%)	13 (40.6%)	59 (34.9%)	NS
HPV-associated cancers and precancerous lesions in history	36 (17.9%)	8 (25.0%)	28 (16.6%)	NS
Abnormal PAP smear in the history	28 (13.9%)	7 (21.9%)	21 (12.4%)	NS
Abnormal HPV smear in the history	16/200 (8.0%)	8/31 (25.8%)	8 (4.7%)	***
Vaccinated against HPV	25 (12.4%)	7 (21.9%)	18 (10.7%)	NS
Before first intercourse	17/25 (68.0%)	4/7 (57.1%)	13/18 (72.2%)	NS
Before transplantation	6/25 (24.0%)	1/7 (14.3%)	5/18 (27.8%)	NS
**Currently taking immunosuppression**	196 (97.5%)	32 (100.0%)	164 (97.0%)	NS
**Number of immunosuppressants**				NS
None	5 (2.5%)	0 (0.0%)	5 (3.0%)	
Single drug	45 (22.4%)	9 (28.1%)	36 (21.3%)	
Multiple drugs	151 (75.1%)	23 (71.9%)	128 (75.7%)	
CNI (Tacrolimus, Cyclosporin A)	177 (88.1%)	29 (90.6%)	148 (87.6%)	NS
mTOR-Inhibitor (Everolimus, Sirolimus)	55 (27.4%)	11 (34.4%)	44 (26.0%)	NS
Antiproliferative substances (Azathioprine, Mycophenolate Mofetil, Mycophenolsäure)	91 (45.3%)	13 (40.6%)	78 (46.2%)	NS
Prednisolone	68 (33.8%)	13 (40.6%)	55 (32.5%)	NS
Other immunosuppressants	2 (6.2%)	8 (4.7%)	10 (5.0%)	NS
**Number of other drugs taken**				**
None	3 (1.5%)	3 (9.4%)	0 (0.0%)	
0–2	65 (32.3%)	11 (34.4%)	54 (32.0%)	
3–5	51 (25.4%)	4 (12.5%)	47 (27.8%)	
>5	82 (40.8%)	14 (43.8%)	68 (40.2%)	
Induction therapy (Basiliximab, ATG, prednisolone)	90/137 (65.7%)	16/24 (66.7%)	74/113 (65.5%)	NS
Multiple transplantations	38 (18.9%)	6 (18.8%)	32 (18.9%)	NS
Rejection	95 (47.3%)	17 (53.1%)	78 (46.2%)	NS
**Age at first intercourse**				
Median (Range)	17.0 (12.0, 39.0)	16.0 (13.0, 24.0)	17.0 (12.0, 39.0)	*
Number of sexual partners				***
Nmiss	1	0	1	
Median (Range)	4.0 (0.0, 30.0)	7.0 (1.0, 30.0)	3.0 (0.0, 25.0)	
0–1	40/200 (20.0%)	1 (3.1%)	39/168 (23.2%)	***
2–5	92/200 (46.0%)	12 (37.5%)	80/168 (47.6%)	
6–10	50/200 (25.0%)	12 (37.5%)	38/168 (22.6%)	
>10	18/200 (9.0%)	7 (21.9%)	11/168 (6.5%)	
Number of sexual partners since Tx				
Median (Range)	1.0 (0.0, 15.0)	1.5 (0.0, 10.0)	1.0 (0.0, 15.0)	***
0–1	151 (75.1%)	16 (50.0%)	135 (79.9%)	***
2–5	41 (20.4%)	11 (34.4%)	30 (17.8%)	
6–10	8 (4.0%)	5 (15.6%)	3 (1.8%)	
>10	1 (0.5%)	0 (0.0%)	1 (0.6%)	
Anal hrHPV **infection**	40/197 (20.3%)	22 (68.8%)	18/165 (10.9%)	***

ATG = Anti-thymocyte globulin, CNI = Calcineurin inhibitors, HPV = human papillomavirus, hrHPV = high-risk human papillomavirus, mTOR-inhibitor = mammalian target of rapamycin- inhibitor, n = number, nmiss = number of missing information, Tx = Transplantation, NS = not significant, * = *p* < 0.05, ** = *p* < 0.01, *** = *p* < 0.001

**Table 2:** Comparison of immunosuppressive treatment of liver transplant recipients, kidney transplant recipients and transplant recipients who received a simultaneous liver and kidney transplantation

	Kidney-Tx (*N* = 98)	Liver-Tx (*N* = 93)	Combined Tx (*N* = 10)	*p*-value
Years since first transplantation Median (Range)	9.0 (1.0, 42.0)	10.0 (1.0, 30.0)	7.0 (1.0, 13.0)	NS
Multiple transplantations	21 (21.4%)	17 (18.3%)	0 (0.0%)	NS
Number of transplantations Median (range)	1.0 (1.0, 5.0)	1.0 (1.0, 3.0)	2.0 (2.0, 2.0)	***
Rejection	43 (43.9%)	50 (53.8%)	2 (20.0%)	NS
Number of immunosuppressive drugs				***
none	5 (5.1%)	0 (0.0%)	0 (0.0%)	
Single	9 (9.2%)	36 (38.7%)	0 (0.0%)	
Multiple	84 (85.7%)	57 (61.3%)	10 (100.0%)	
CNI (Tacrolimus, Cyclosporin A)	79 (80.6%)	89 (95.7%)	9 (90.0%)	**
mTOR-Inhibitora (Everolimus, Sirolimus)	25 (25.5%)	22 (23.7%)	8 (80.0%)	***
Antiproliferative substances (Azathioprine, Mycophenolate Mofetil, Mycophenolsäure)	55 (56.1%)	35 (37.6%)	1 (10.0%)	***
Prednisolone	50 (51.0%)	17 (18.3%)	1 (10.0%)	***
Others	9 (9.2%)	0 (0.0%)	1 (10.0%)	**
Induction therapy (Basiliximab, Anti-thymocyte globulin, prednisolone)	65/76 (85.5%)	20/54 (37.0%)	5/7 (71.4%)	***
Cervical hrHPV	17 (17.3%)	14 (15.1%)	1 (10%)	NS
Anal hrHPV	21/95 (22.1%)	17/92 (18.5%)	2 (20.0%)	NS

CNI = Calcineurin inhibitors, HPV = human papillomavirus, hrHPV = high-risk human papillomavirus, mTOR-inhibitor = mammalian target of rapamycin- inhibitor, N = number, Tx = Transplantation, NS = not significant, * = *p* < 0.05, ** = *p* < 0.01, *** = *p* < 0.001

### Immunosuppressive treatment

Overall, 97.5% of the patients were currently taking immunosuppressants, with most (75%) being on a calcineurin inhibitor (CNI) based immunosuppressive regimen consisting of more than two drugs. A comparison of immunosuppression and HPV risk factors is shown in Table 1. Neither the number of immunosuppressants nor the type of immunosuppression was significantly associated with hrHPV infection. Other transplant-specific variables, such as duration of immunosuppressive treatment, pretransplant immunosuppressant use, or graft rejections, did not correlate with hrHPV infection. A comparison of kidney and liver transplant recipients showed differences in immunosuppressive therapy (see Table 2). Compared to liver transplants, more kidney recipients took at least two immunosuppressants and had higher induction therapy rates (85.5% vs. 37%). However, cervical hrHPV prevalence showed no significant difference. Additionally, risk factors like sexual partners and age at first intercourse didn’t differ between kidney and liver recipients.

Risk factors associated with increased HPV prevalence in the general population, i.e. sexual behaviour and younger age, are confirmed in transplanted patients in a multivariable logistic regression model, with adjusting for transplantation, nicotine use and age of first intercourse (see Table 3). HrHPV-positive women were, on average, one year younger at the time of first sexual intercourse (*p* = 0.025) and had more than twice as many sexual partners as HPV- negative patients (*p* < 0.001). Likewise, the number of sexual partners since the first transplant was significantly higher among hrHPV-positive patients (*p* < 0.001).

**Table 3:** Multiple logistic regression of influencing factors on cervical HrHPV positvity

	HPV cervix		
*Predictors*	*Odds Ratios*	*CI*	*p-value*
Liver Tx	0.90	0.39–2.08	0.803
Kidney or combined Tx	1.00	0.05–7.12	0.998
Number of sexual partners	1.13	1.05–1.21	**0.001**
smoking	1.30	0.36–3.93	0.663
Age (in years)	0.97	0.94–0.99	**0.018**
Age at first intecourse	0.93	0.78–1.06	0.340
Observations	200		
R^2^ Tjur	0.113		

Tx = transplantation, CI = confidence interval

### HPV vaccination

In total, 12.4% (25/201) of patients had prior HPV vaccination. Among them, 80% (20/25) were under 30, while only 2.9% (5/173) of those over 30 were vaccinated (*p* < 0.0001). Most patients were vaccinated before their first sexual intercourse (17/25), and six were vaccinated before transplantation. The number of vaccinated patients was higher among HPV-positive patients compared with HPV-negative patients (see Table 1). Three HPV-positive women were vaccinated after their first sexual intercourse and six after their transplantation.

### Follow-up

The participation rate in the follow-up offered 6 to 18 months after the initial HPV test for cervical hr-HPV positive patients was 91%, with 29 out of 32 patients returning. The positive hr-HPV result was confirmed in 25 cases. In 52% (13/25), more than one HPV type was determined by genotyping. A total of 14 different HPV-Types with oncogenic potential were detected in the cervical site (HPV 16, 18, 31, 33, 39, 45, 51, 52, 56, 58, 59, 66, 68, 73). High-grade squamous intraepithelial lesion (HSIL) was detected in four patients during follow-up in the dysplasia unit (1x CIN2, 2x CIN3, 1x VaIN3). Three out of four patients with precancerous lesions had normal pap smears (NILM).

**Fig. 2:**
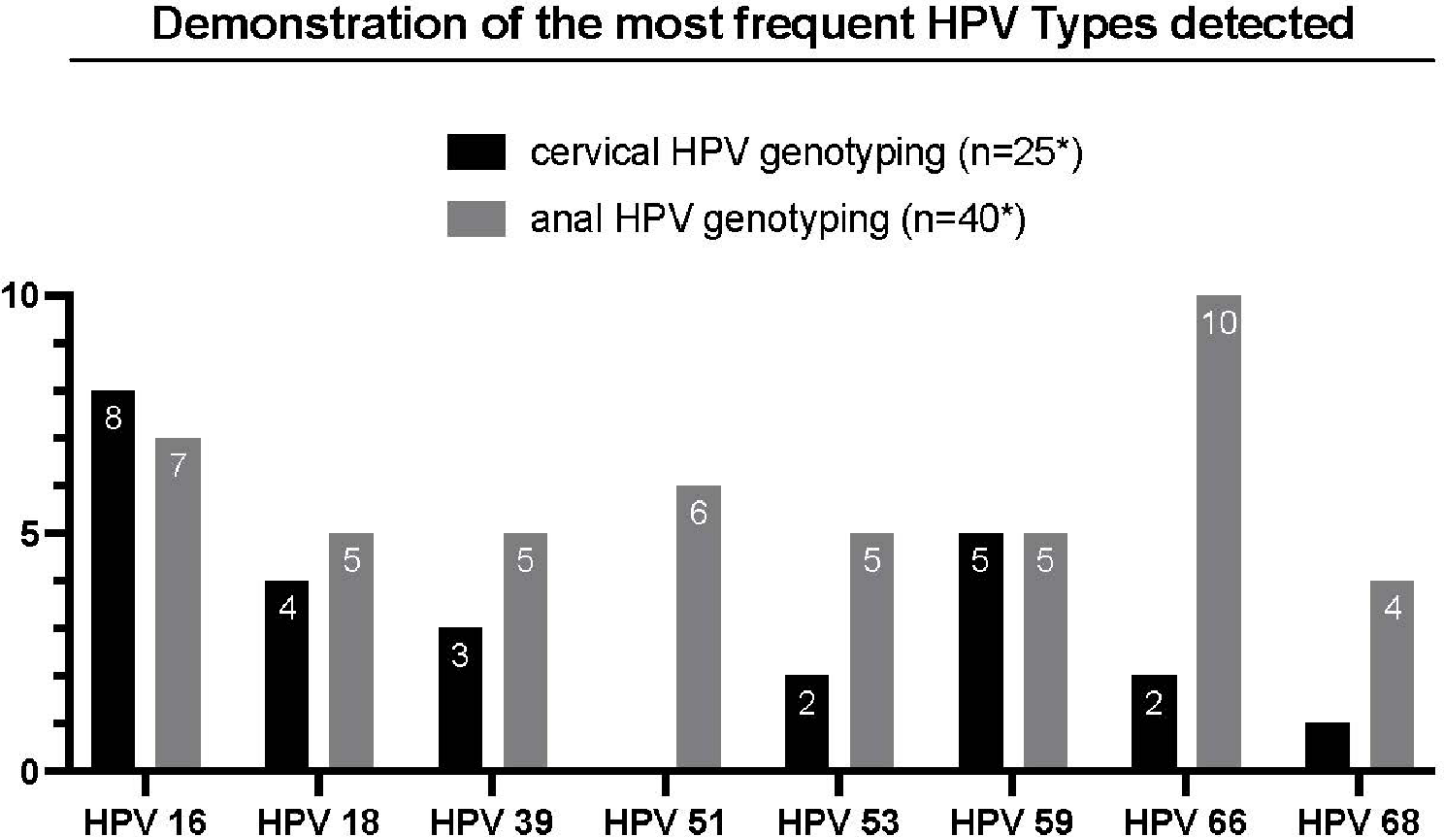
* This refers to the number of tested patients; multiple HPV types can occur in a single patient. HPV = Human papillomavirus

In anal swab specimens more than one HPV genotype was detected in 52.5% of patients, and 11 different high-risk oncogenic genotypes were identified (HPV 16, 18, 31, 39, 45, 51, 52, 56, 58, 59, 68). The most common HPV-types are shown in Fig. 2.

### Anal and cervical co-infection

For further investigation of co-infection in cervical hrHPV-positive patients, genotyping was performed at follow-up, whereas anal HPV testing involved genotyping at baseline and follow-up, and one positive test was considered sufficient for hrHPV positivity. All detected HPV types were included in the evaluation of co-infection, regardless of the time of examination. In patients with cervical hrHPV positivity, anal co-infection was detected in 68,8% (22/32), whereas in cervical HPV-negative patients, only 10.9% (18/165) had an anal hrHPV infection. In 18 of 23 patients, cervical and anal genotyping was available to compare HPV genotypes. Fifteen of these 18 patients were positive for more than one HPV type. There was a strong association at the HPV-specific level: 78% (14/18) of patients exhibited at least one concordant hrHPV type, while 22% (4/18) displayed different hrHPV genotypes. The highest concordance rate was found for HPV 16, with all 6 out of 6 patients (100%) showing a coinfection in the anal swab specimens.

## Discussion

This study is one of the largest studies investigating genitoanal hrHPV prevalence in female transplant recipients published so far. Overall, the observed cervical hrHPV prevalence was 15.9%, and the anal hrHPV prevalence was 20.3%. Compared to the general population worldwide, cervical hrHPV prevalence is more than twice as high in transplant recipients (15.9% vs. 8.1%) [19]. In particular, patients older than 30 are at increased risk for hrHPV infection, with a prevalence of 14.5% compared to 6.2% in the general population in Germany, while transplanted women younger than 30 show no increased hrHPV prevalence [20, 21]. These findings are aligned with other larger studies that observed a cervical hrHPV prevalence of 15.2% and 17.4% in solid organ transplant recipients [14, 15].

The HPV prevalence data for patients under 30 used in this study were obtained from a 2010 publication. To the author’s knowledge, there is no more recent data on hrHPV prevalence in the German population under 30 available. Yet, US trends show vaccination reduces HPV prevalence in young women [22]. Obtaining comparable data for the German population in the future could help evaluate hrHPV prevalence in young, transplanted patients compared to vaccinated women.

Encouragingly, the cervical screening participation rate was over 90%. Four out of 29 patients had undetected HSIL with three of them being over 30 years old. This raises the question of whether cervical screening should be intensified in transplant recipients. In our study, three out of four patients with precancerous lesions had normal pap smears. Currently, repeating co-testing after one year is recommended for women in Germany with hrHPV positivity and normal cytology. Immunosuppressed patients with hrHPV infection might benefit from direct referral to colposcopy considering the high probability of hrHPV persistence and increased prevalence of precancerous lesions [4].

Our findings suggest that neither the time since transplantation nor the type of iatrogenic immunosuppression were associated with increased hrHPV prevalence. Based on our results, the only significant transplantation-specific risk factor for HPV infection appears to be immunosuppression itself. This is consistent with the findings of other studies in transplanted patients, which showed that duration and intensity were only weak determinants for increased hrHPV prevalence [14, 23]. The significant influence of known risk factors for HPV like sexual behavior, was confirmed by the results of our study. Still, these risk factors lead to a higher prevalence of hrHPV than in the general population. The reasons might be a longer persistence of HPV and a lower clearance rate in immunosuppressed patients leading to a higher risk for precancerous lesions. This highlights the need for regular screening in a high-risk group to detect the development of precancerous lesions on time. Also, patients should be informed about the risk factors and transmission of HPV infection and the development of HPV associated diseases, as only 63% of patients were aware of their increased cancer risk.

Compared to cervical hrHPV prevalence, anal hrHPV prevalence is less well studied. Lin et al. reported an anal HPV prevalence of 9% for women who are cervical HPV negative, compared to a 43% co-infection rate for cervical hrHPV positive women [3]. Our study described an even higher rate of co-infection at cervical and anal sites of 69% in immunosuppressed women. The hrHPV prevalence in women who were cervical HPV negative was 10.9% and is comparable to the results of Lin et al.

Recently, it was shown that the prevalence of anal HSIL is higher in patients with cervical hrHPV positivity [3]. However, the transition from infection to dysplasia can take decades, and most anal HPV infections are thought to clear on their own. This is especially true for immunocompetent patients. HIV-infected patients have been shown to be less likely to clear HPV than HIV-negative patients [24]. The increased risk of anal cancer in transplanted patients is well known. The risks appear to increase with the number of years since transplantation [5]. Given the high prevalence of anal hrHPV infections, the patient groups at the highest risk should be identified to initiate targeted secondary prevention. Women with cervical HPV16 infection over 45 years have a comparable anal cancer risk to HIV-positive patients, where anal cancer screening is recommended [3]. Our findings suggest cervical hrHPV positivity could be a surrogate marker for anal hrHPV co-infection that may help stratify transplanted patients and initiate screening. The extent to which other hrHPV types besides HPV16 can also serve as surrogate markers and how many cases of hrHPV infection lead to anal carcinoma in transplanted patients still needs to be investigated in further studies. Current guidelines recommend screening 10 years post-transplantation using digital rectal exam combined with anal cytology and/or anal HPV testing, which may be appropriate for patients without a history of HPV-related dysplasia or cancer [18]. However, transplant recipients with known HPV-related disease face a significantly higher risk of developing anal HPV associated disease including cancer [25]. In such cases, anal cancer screening should be recommended regardless of the time elapsed since transplantation. High resolution anoscopy should be performed in case of abnormal cytology or hrHPV positivity [18].

It could also be considered whether anal HPV testing should be integrated into cervical screening protocols, given its feasibility. With an estimated anal high-risk HPV (hrHPV) prevalence of approximately 20%, around one in five patients would qualify for referral to high-resolution anoscopy. However, the potential benefit of this strategy in terms of earlier detection of anal intraepithelial neoplasia (AIN) and anal carcinoma in transplant recipients remains uncertain and requires further evaluation in prospective studies before implementation can be recommended. This might be even more relevant for younger patients as our fings suggest that age-related differences in hrHPV prevalence may be more pronounced for anal than for cervical sites in this population.

The overall vaccination rate was only about 12%. One reason for the low vaccination rate in our study is the high age of the participating patients. Age was significantly lower in vaccinated patients (71.4% ≤30 years vs. 2.9% >30 years).

However, our results indicate that sexual behaviour is the leading risk factor for hrHPV infection regardless of age. Because the prevalence of hrHPV was increased in the age group over 30 years, vaccination of this patient group beyond 18 years can be discussed. In addition, vaccination rates were higher in hrHPV-positive women than in HPV-negative women. One reason for this is undoubtedly the younger average age of the hrHPV-positive cohort. Also, most of the HPV-positive patients were vaccinated after transplantation. Studies suggest that vaccination before transplantation is beneficial for immune response, so vaccination before transplantation should be considered [26].

Although the cross-sectional design limits definitive causal inference, cautious interpretation of the observed associations remains valuable, given the limited number of studies conducted in this patient group. However, the small sample size and lack of longitudinal data highlight the need for further research to conclusively assess influencing factors, such as variations in immunosuppressive therapy regimens. Unfortunately, following up on the number of anal (pre)malignancies was impossible, so risk factors related to anal carcinomas could not be evaluated. Two different HPV tests were used for cervical and anal testing (Cobas vs. Anyplex), but both tests show comparable sensitivity and specificity for detecting cervical HPV infections and are recommended for cancer screening [27]. To the authors’ knowledge, there have been no extensive studies conducted to date on the sensitivity and specificity of HPV tests for the detection of anal intraepithelial neoplasia (AIN) and their comparability with one another. Still, using Anyplex Seegene for anal hrHPV detection is common clinical practice.

It should also be considered that many patients received an HPV test for the first time during the study since HPV testing was not included in regular screening in Germany until 2020. Some of the information evaluated here on sexual behavior, HPV vaccination status and parts of medical history (e.g. HPV-associated diseases) were provided only by patients. Verification of the information using medical records was not always possible.

## Conclusions

Immunosuppressed patients represent a high-risk group for developing anogenital dysplasia and cancer. The risk of harboring an hrHPV infection is increased regardless of the immunosuppressive regimen. Based on our results, adjustment of current screening in this high-risk group can be discussed. If cervical hrHPV is detected, a colposcopic examination should be performed independently of cytology because of the high risk for HPV-dependent disease. Cervical hrHPV positivity can also serve as a surrogate marker for targeted anal screening, especially in the case of HPV16 detection, and possibly regarding other hr-HPV genotypes. Given the high prevalence of hrHPV, particularly in patients over 30, HPV vaccination should be considered, ideally before transplantation.

## Abbreviations

AIN: Anal intraepithelial neoplasia
CIN: Cervixal intraepithelial neoplasia
CNI: Calcineurininhibitor
HPV: Human papillomavirus
hrHPV: High-risk human papillomavirus
HSIL: High-grade squamous intraepithelial lesion
lrHPV: Low risk human papillomavirus
VaIN: Vaginal intraepithelial neoplasia
